# Supplementary material for: Bayesian refinement of protein structures and ensembles against SAXS data using molecular dynamics
Source: PLoS Comput Biol. 2017 Oct 18;13(10):e1005800. doi: 10.1371/journal.pcbi.1005800 (PMC5662244; doi:10.1371/journal.pcbi.1005800)
Supplement: S1 Archive — (BZ2) [file pcbi.1005800.s011.tar.bz2 › gromacs_4.62_bayesian_saxs_refi/share/html/online/g_spatial.html]

g\_spatial


| g\_spatial Main Table of Contents |

**VERSION 4.6  
Sat 19 Jan 2013**

---

### Description

g\_spatial calculates the spatial distribution function and
outputs it in a form that can be read by VMD as Gaussian98 cube format.
This was developed from template.c (GROMACS-3.3).
For a system of 32,000 atoms and a 50 ns trajectory, the SDF can be generated
in about 30 minutes, with most of the time dedicated to the two runs through
trjconv that are required to center everything properly.
This also takes a whole bunch of space (3 copies of the .xtc file).
Still, the pictures are pretty and very informative when the fitted selection is properly made.
3-4 atoms in a widely mobile group (like a free amino acid in solution) works
well, or select the protein backbone in a stable folded structure to get the SDF
of solvent and look at the time-averaged solvation shell.
It is also possible using this program to generate the SDF based on some arbitrary
Cartesian coordinate. To do that, simply omit the preliminary trjconv steps.
USAGE:
1. Use ndx.html">make\_ndx to create a group containing the atoms around which you want the SDF
2. trjconv -s a.tpr -f a.xtc -o b.xtc -center tric -ur compact -pbc none
3. trjconv -s a.tpr -f b.xtc -o c.xtc -fit rot+trans
4. run g\_spatial on the .xtc output of step #3.
5. Load grid.cube into VMD and view as an isosurface.
**Note** that systems such as micelles will require trjconv -pbc cluster between steps 1 and 2
WARNINGS:  
The SDF will be generated for a cube that contains all bins that have some non-zero occupancy.
However, the preparatory -fit rot+trans option to trjconv implies that your system will be rotating
and translating in space (in order that the selected group does not). Therefore the values that are
returned will only be valid for some region around your central group/coordinate that has full overlap
with system volume throughout the entire translated/rotated system over the course of the trajectory.
It is up to the user to ensure that this is the case.
BUGS:  
When the allocated memory is not large enough, a segmentation fault may occur. This is usually detected
and the program is halted prior to the fault while displaying a warning message suggesting the use of the -nab (Number of Additional Bins)
option. However, the program does not detect all such events. If you encounter a segmentation fault, run it again
with an increased -nab value.
RISKY OPTIONS:  
To reduce the amount of space and time required, you can output only the coords
that are going to be used in the first and subsequent run through trjconv.
However, be sure to set the -nab option to a sufficiently high value since
memory is allocated for cube bins based on the initial coordinates and the -nab
option value.

### Files

| option | filename | type | description |
| --- | --- | --- | --- |
| **-s** | topol.tpr | Input | Structure+mass(db): tpr tpb tpa gro g96 pdb |
| **-f** | traj.xtc | Input | Trajectory: xtc trr trj gro g96 pdb cpt |
| **-n** | index.ndx | Input, Opt. | Index file |

### Other options

| option | type | default | description |
| --- | --- | --- | --- |
| **-[no]h** | bool | no | Print help info and quit || **-[no]version** | bool | no | Print version info and quit || **-nice** | int | 0 | Set the nicelevel || **-b** | time | 0 | First frame (ps) to read from trajectory || **-e** | time | 0 | Last frame (ps) to read from trajectory || **-dt** | time | 0 | Only use frame when t MOD dt = first time (ps) || **-[no]w** | bool | no | View output .xvg, .xpm, .eps and .pdb files || **-[no]pbc** | bool | no | Use periodic boundary conditions for computing distances || **-[no]div** | bool | yes | Calculate and apply the divisor for bin occupancies based on atoms/minimal cube size. Set as TRUE for visualization and as FALSE (-nodiv) to get accurate counts per frame || **-ign** | int | -1 | Do not display this number of outer cubes (positive values may reduce boundary speckles; -1 ensures outer surface is visible) || **-bin** | real | 0.05 | Width of the bins (nm) || **-nab** | int | 4 | Number of additional bins to ensure proper memory allocation |

---

http://www.gromacs.org  
gromacs@gromacs.org
